# Occurrence and characterization of viruses infecting Amorphophallus in Yunnan, China

**DOI:** 10.1038/s41598-024-63477-y

**Published:** 2024-06-05

**Authors:** Jiahong Dong, Ting Zhu, Rui Lv, Kun Dong, Yu Li, Boxin Zhang, Lizhen Zhang, Yongdui Chen, Xiangao Yin, Lei Zhang, Jianqing Yin, Jun Lu, Dehui Xi, Kuo Wu

**Affiliations:** 1 Institute of Medicinal Plant Cultivation, School of Chinese Materia Medica and Yunnan Key Laboratory of Southern Medicinal Resource, Kunming, Yunnan China; 2 Key Laboratory of Bio-Resource and Eco-Environment of Ministry of Education, College of Life Sciences, Sichuan University, Chengdu, China; 3 Fuyuan Konjac Institute, Yunnan Academy of Agricultural Sciences, Qujing, Yunnan China; 4 Biotechnology and Germplasm Resources Institute, Yunnan Academy of Agricultural Sciences, Kunming, Yunnan China; 5 Seed Management Station of Fuyuan County, Qujing, Yunnan China

**Keywords:** Pathogens, Virology

## Abstract

Viral diseases are becoming an important problem in Amorphophallus production due to the propagation of seed corms and their trade across regions. In this study, combined-High-Throughput Sequencing, RT-PCR, electron microscopy, and mechanical inoculation were used to analyze virus-like infected Amorphophallus samples in Yunnan province to investigate the distribution, molecular characterization, and diversity and evolution of Amorphophallus-infecting viruses including three isolates of dasheen mosaic virus and three orthotospoviruses: mulberry vein banding associated virus (MVBaV), tomato zonate spot virus (TZSV) and impatiens necrotic spot virus (INSV). The results showed that DsMV is the dominant virus infecting Amorphophallus, mixed infections with DsMV and MVBaV to Amorphophallus were quite common in Yunnan province, China. This is the first report on infection of Amorphophallus with MVBaV, TZSV, and impatiens necrotic spot virus (INSV) in China. This work will help to develop an effective integrated management strategy to control the spread of Amorphophallus viral diseases.

## Introduction

The genus *Amorphophallus* Blume ex Decne comprises 242 accepted species of domesticated and wild Amorphophallus (konjac) and is the third largest genus in the *Araceae* family and is mainly distributed in the Tropical and Southern Africa, Madagascar, and Tropical and Subtropical Asia to Northern Australia^1^. The bulb of *Amorphophallus* is rich in glucomannan, which is widely used in food, medicine, and the chemical industry^2^. Because the glucomannan content in the bulbs of *A. konjac* and *A. albus* is approximately 50–65% dry weight, these two species have been cultivated annually on about 140,000 hectares (ha) in Yunnan, Sichuan, Guizhou provinces of southwest China as economically important crops^3^. Here it contributes to 60–70% of the global Konjac production. Yunnan province is the main cultivator of Amorphophallus, where planting areas make up about 36% of China. In 2020, approximately 10,000 ha of these two species were planted only in Fuyuan County of Yunnan province, with an agricultural production value of $150 million, while Amorphophallus seed corms grown in Fuyuan were estimated to account for more than 50% of the Amorphophallus seed corms market in China. However, because of continuous cropping and vegetative propagation, diseases such as bacterial soft rot caused by *Pectobacterium* sp., anthracnose caused by *Colletotrichum* sp. and viral diseases have become the major serious problem in Amorphophallus production, causing a 30–70% reduction in yield^4–6^.

In recent years, field surveys showed that viral diseases could significantly affect the production of konjac, where three viruses have been reported to infect Amorphophallus, including konjac mosaic virus (KoMV) and dasheen mosaic virus (DaMV), which belong to the genus *Potyvirus* of family *Potyviridae*, and cucumber mosaic virus (CMV, genus *Cucumovirus*, family *Bromoviridae*) in Japan^7–9^, India^10^, and China^11,12^. Both KoMV and DaMV are experimentally transmitted by infected sap and, in nature, by several widely distributed aphid species, including *Myzus persicae* and *Aphis gossypii* in a non-persistent manner^7,8^.

Despite its socio-economic importance, little is known about viral diseases associated with konjac in China except for sporadic reports on infections of DsMV and CMV^11,12^. Since Amorphophallus cultivation relay almost exclusively on vegetative propagation, the viruses are transmitted by the seed corm of Amorphophallus and are spread over long distances. Recently, typical viral symptoms including mosaic, necrotic, chlorotic yellow, mottle, stunt, and leaf crinkling were found on Amorphophallus plants in Yunnan province. In this study, a total of 146 Amorphophallus and 14 weeds and other crop plants or leaves with virus-like symptoms were mainly collected during the 2016–2021 growing seasons, and subjected to high-throughput sequencing (HTS), RT-PCR, and/or electron microscopy to determine the presence and identity of the potential viral pathogens.

## Results

### Symptoms and incidence of *Amorphophallus* viral diseases

**Figure 1:**
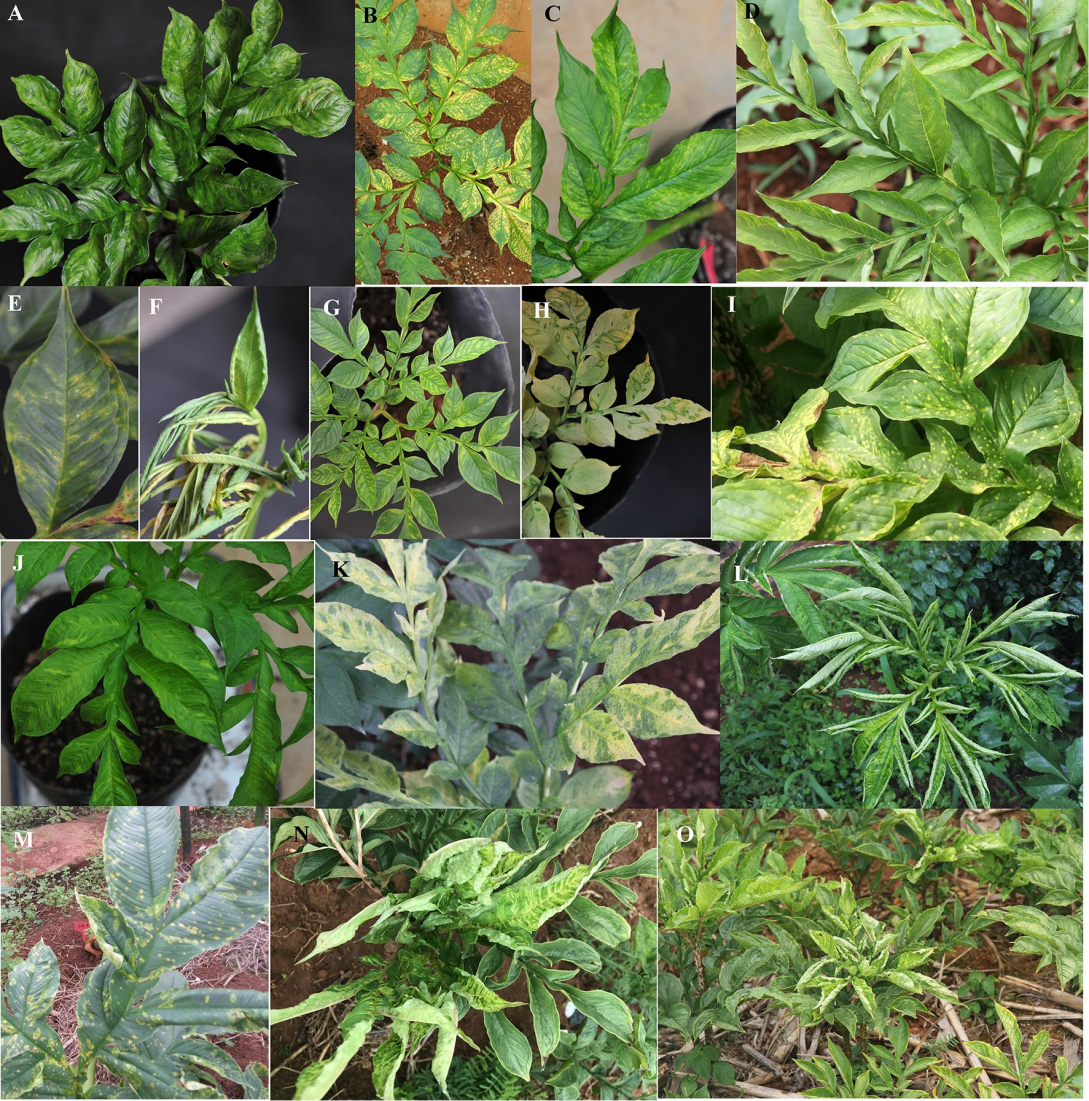
Typical symptoms of viral infection in Amorphophallus leaves. (**A**) Mosaic (Albus-18), (**B**) yellow necrotic spot (Albus-9), (**C**) yellow (Konjac-52), (**D**) chlorotic ringspot (Albus-15), (**E**) chlorotic and necrotic, ringspot (Albus-16), (**F**) leaf roll, chlorotic and wilt (Albus-17), (**G**) mottle and chlorosis, stunting (Kojac-59), (**H**) Bleach and irregular green ringspots (Konjac-57), (**I**) yellow and necrotic ringspot (Konjac-53), (**J**) mosaic (Konjac-43), (**K**) chlorotic mosaic (Konjac-77), (**L**) leaf roll and feather mosaic (Bulbifer-5), (**M**) yellow ringspot (Bulbifer-2), (**N**) feather mosaic (Konjac-91) and (**O**) image of field with more than 20% viral infection prevalence.

The virus-infected Amorphophallu*s* plants exhibited symptoms of leaf mosaic, mottling, chlorotic spot, ringspot, puckering, leaf distorting, shoestringing, and feathery mottle symptoms (Fig. 1), and some infected plants were stunted. The disease incidence estimation mainly carried out at six sites in Fuyuan county, Yunnan province. Number of plants observed in each site for virus-like disease estimation was more than 100. All the virus-like disease symptoms were accounted for disease estimation. The average disease incidence of 15% was observed during July and August every year. The high disease incidence (more than 50%) occurred at a germplasm plantation located at Zhongan town in Fuyuan county where Amorphophallus have been planted for at least ten years. Five percent of the Amorphophallus plants grown from the randomly collected seed corms showed viral symptoms such as leaf mosaic, and necrotic or chlorotic spots on leaves.

### EM examination

**Figure 2:**
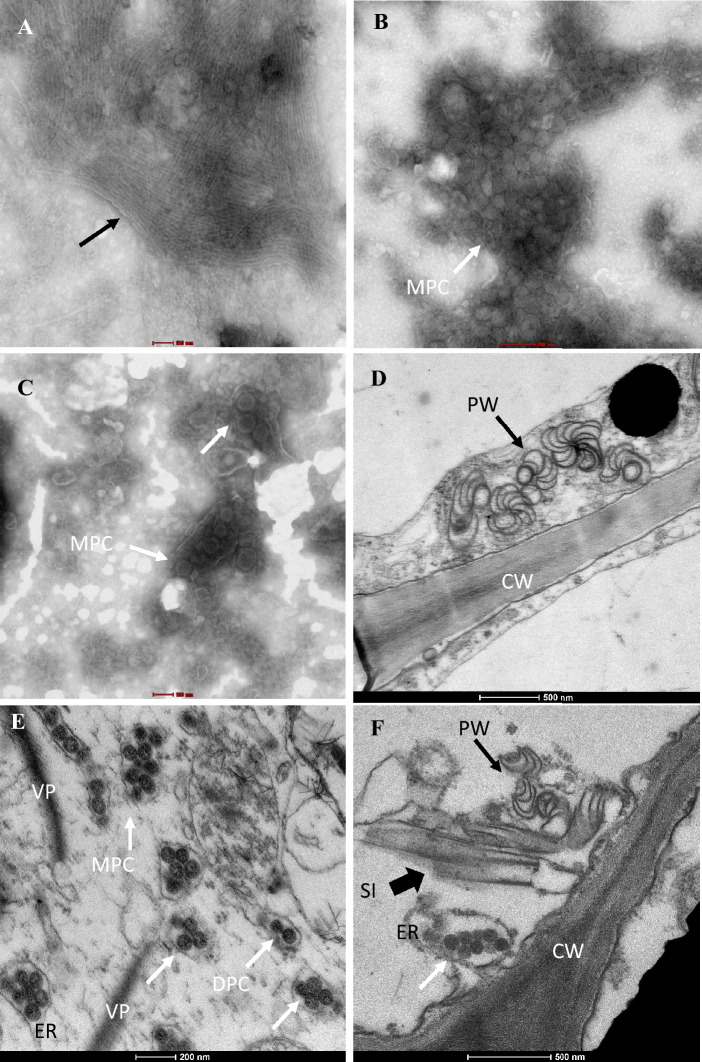
Transmission electron micrographs showing the particles in the saps of the infected leaves by negative preparation, (**A**). The aggregation of DsMV particles (black arrow) in sample Albus-19, (**B**) MVBaV particles clustering (white arrow) in sample Konjac-49 and TZSV partcles clustering (white arrow) in sample Albus-16; the ultrastructure of the leaf tissue of sample Konjac-53 (**D**, **E**) and Albus-13 (**F**), which was co-infected with DsMV and MVBaV, (**D**) showing the pinwheel (PW) inclusion bodies (black arrow) caused by DsMV, (**E**) showing orthotospovirus particle clusters (whitearrow), (**F**) showing mixed infection by DsMV and MVBaV, pinwheel inclusion bodies (black arrow), striped inclusions (SI) (black wide arrow), multiple particle clusters (MPC) (white arrow) enveloped in the membrane vesicle. VP, Viroplasm; CW, cell wall; DPC, double particles clustering.

Electron microscope examination of naturally infected *A. konjac*, *A. albus*, *A. bulbifer*, and weeds revealed the aggregations of flexuous and filamentous particles of 700 nm in length (Fig. 2A) and roughly spherical enveloped virion particles measuring 70–100 nm in diameter (Fig. 2B, C). The pinwheel and striped inclusion bodies, which often represent the typical cytopathological features of potyvirus infections, were observed in the cytoplasm of the DsMV-infected samples (Fig. 2D and F). We also observed viral particles aggregated in the swollen of endoplasmic reticulum forming specific clustering patterns of the orthotospovirus particles, single particle, double particles clustering, triple particles clustering and multiple particles clustering (Fig. 2E and F)^13^. In some cells, the globular viroplasm (VP) were observed (Fig. 2E). By contrast, no viral particles or inclusion bodies were observed in a virus-negative sample. Among 160 samples, the 122 samples were examined by EM, 106/122 samples had filamentous particles in the crude leaf extracts, 83/122 samples had orthotospovirus-like particles and 69/122 showed mixed infection (Table S1).

### High-throughput sequencing and sequence assembly

In this study, four Amorphophallus samples, Konjac-sym, Konjac-asym, Albus-sym, and Albus-asym, were analyzed with HTS on an Illumina MiSeq platform and generated a total of 61,277,370, 88,345,484, 133,491,322, and 82,237,592 useable reads, respectively (Table S2). Sample Konjac-sym showed typical mosaic and necrotic symptoms on the leaves of A. *konjac*, while sample Konjac-asym showed symptomless on the leaves of A. *konjac*. Sample Albus-sym showed typical mosaic and necrotic symptoms on the leaves of *A. albus*, while Albus-asym showed symptomless on the leaves of *A. albus*. A total of 56,509 and 50,868 contigs which is more than 300 bp were generated from Konjac-sym and Konjac-asym, respectively. For Albus-sym and Albus-asym, 158,104 and 159,756 contigs were generated, respectively. The contigs from each of the four Amorphophallus were analyzed by the BLASTx search of the NCBI databases. The search results revealed viral contigs identified in two symptomatic samples: Konjac-sym and Albus-sym included those of DsMV (a potyvirus), tomato zonate spot virus (TZSV), and mulberry vein banding associated virus (MVBaV), where the latter two viruses belong to the orthotospoviruses (family *Tospoviridae*). No contigs were mapped to virus sequences in the HTS data of two asymptomatic samples: Konjac-asym and Albus-asym. This result matched EM examination (Table S1).

### Infection of TZSV, and MVBaV to *A. konjac*

**Figure 3:**
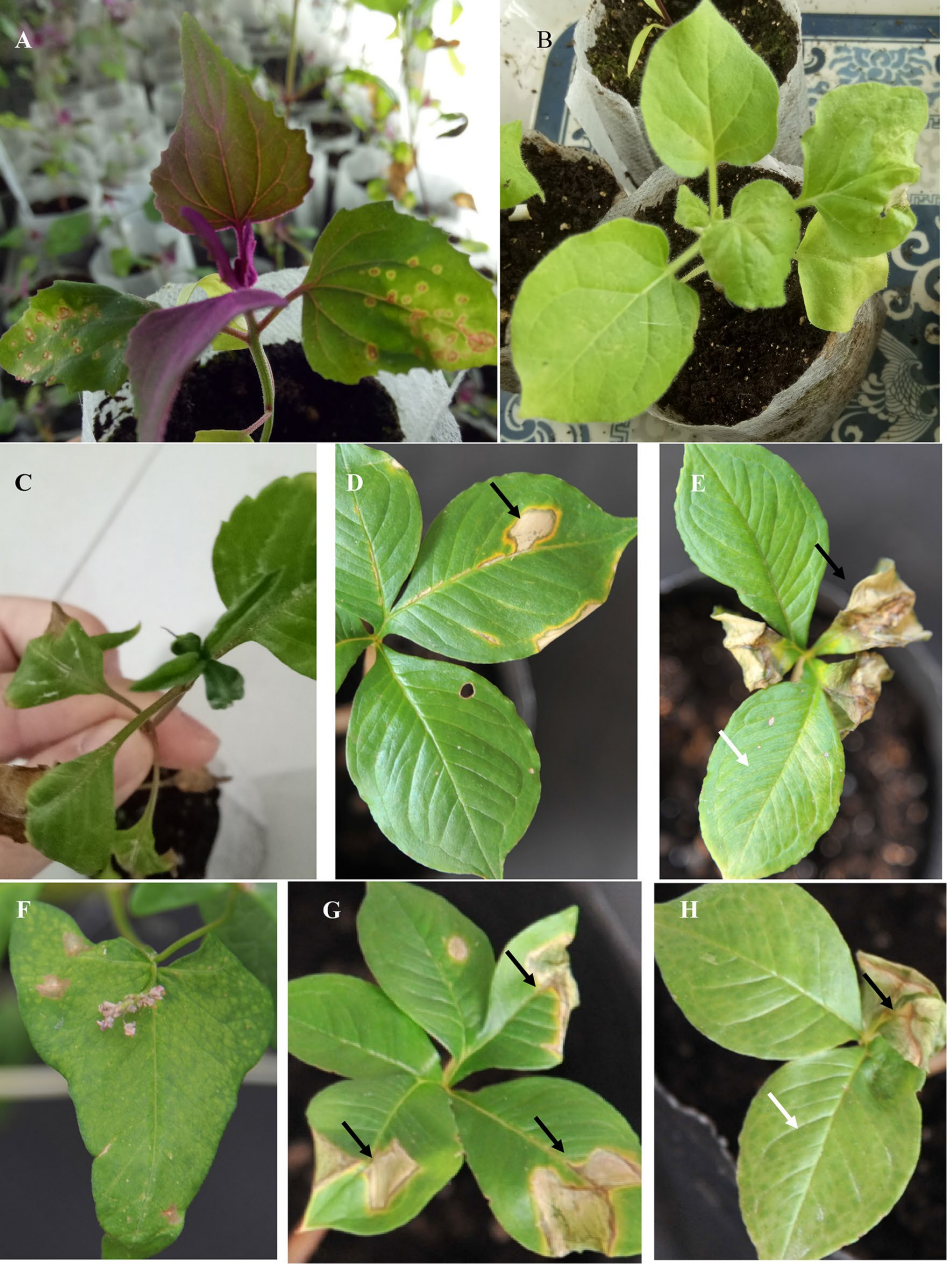
Infection of *A. konjac* and other plants with two orthotospoviruses through mechanical inoculation. (**A**) Lesions on the MVBaV-inoculated leaves of *Chenopodium amaranticolor* at seven days post inoculation (dpi), (**B**) chlorosis and necrotic spot on the MVBaV-inoculated leaves of *Nicotiana benthamiana* at 7 dpi, (**C**) leaf distorting on the MVBaV-inoculated leaves of *Bidens Pilosa* at 7 dpi, (**D**) unsuccessful infection for MVBaV-inoculated *A. konjac* plant at 20 dpi, there are rubbed spots (arrow) on the inoculated leaves, and no virus was detected from this plant by RT-PCR, (**E**) the inoculated leaves are fully withered (arrow), and chlorosis and necrotic spots (white arrow) on systemic leaves of a MVBaV-inoculated *A. konjac* plant at 20 dpi and positive result for virus detection, (**F**) chlorotic spots on system leaf of TZSV-inoculated buckwheat at 7 dpi, (**G**) unsuccessful infection for TZSV-inoculated *A. konjac* plant at 20 dpi, there are rubbed withered spots on the inoculated leaves, and no virus was detected from this plant by RT-PCR, (**H**) the inoculated leaves are fully withered, mottle and chlorosis on the system leaves of TZSV-inoculated *A. konjac* plant at 7 dpi.

Specifically, DsMV was detected in two symptomatic samples Konjac-sym and Albus-sym, while TZSV and MVBaV were only detected in Albus-sym. Results from RT-PCR analysis indicated these three viruses could infect *A. konjac* and *A. albus*. The sequences of each of the amplicons matched the corresponding sequences obtained by HTS with 100% nt sequence identity. The infection of TZSV, and MVBaV was verified by fulfilling Koch’s Postulates (Fig. 3) and inoculating healthy *A. konjac* and *A. albus* seedlings with infected plant sap. However, the successful infection of both TZSV and MVBaV in Amorphophallus through mechanical transmission was less than 20%. Except for rubbed spots, mosaic was the main symptom in the DsMV-inoculated *A. konjac* plants at 20 days post-inoculation. For two orthotospoviruses (TZSV-Konjac and MVBaV-Konjac), local lesions were observed on the inoculated leaves of *Chenopodium amaranticolor* at seven days post inoculation (dpi) as well as what other orthotospoviruses caused in this lesion host (Dong et al.^32^) (Fig. 3A), chlorosis and necrotic spots were observed on the inoculated and systemic leaves of *Nicotiana benthamiana* since 5 dpi (Fig. 3B). MVBaV-Konjac caused Leaf distorting of *Bidens Pilosa* at 7dpi (Fig. 3C). Both TZSV-Konjac and MVBaV-Konjac caused the inoculated leaves fully withered, motting and chlorosis on the systemic leaves (Fig. 3E, H). While unsuccessfully infected *A. konjac* plants (Mock) showed rubbed spots (arrow) on the inoculated leaves, no viral disease symptoms on the systemic leaves (Fig. 3D, G), and no virus was detected from this plant by RT-PCR.

### Dasheen mosaic virus genome assembly and complete genome sequence comparisons

Using BLASTX searches on the NCBI RefSeq protein database to analyze the contigs (konjac, n = 58,294; albus, n = 202,100), one contig of 10,036 bp in length from Konjac-sym and another one of 9978 bp in length from Albus-sym were homologous to the complete coding sequences for the polyprotein of DsMV. This confirms the infection of the samples by DsMV. The full genome of these two DsMV isolates (DsMV Konjac-sym from Konjac-sym and DsMV Ablus-sym from Ablus-sym) were completed according to the methodology^14,15^. The full genome of the DsMV AK-83 isolate was also obtained by genome walking with RT-PCR amplification.

The nucleotide acid sequence of DsMV Konjac-sym was determined to be 10,049 nt in length (Acc. no. OR911345) and had 5′- and 3′-nontranslated regions (NTRs) of 167 and 248 nt, respectively. The full genome sequence of DsMV Ablus-sym was 9972 nt in length (OR911343) and had 5′- and 3′-NTRs of 167 and 260 nt, respectively. The complete sequence of DsMV AK-83 genome (OR911344) was determined to be 10,011 nt, and had 5′- and 3′-NTRs of 167 and 248 nt, respectively. All three isolates possessed the typical genome organizations of DsMV and other potyviruses, made up of one open reading frame (ORF) that encodes a large polyprotein of 3205, 3177, and 3199 amino acid residues, respectively.

The genome sequences of the three isolates, DsMV Konjac-sym, Albus-sym, and AK-83, were 76.3–94.3% genetically similar, with DsMV-Albus-sym isolate being the most divergent (Table S3). The DsMV-Albus-sym genome sequence had a larger identity range of 74.6% (isolate VanMV CI, KX505964.1) to 77.9% (DsMV isolate Et14 MG602232.2) at the nucleotide level and 84.55–88.54% at the amino acid level. This range was wider than that of DsMV-Konjac-sym and AK-83, which showed identities ranging from 79.9 to 89.7% at the nucleotide level and 86.6–93.49% at the amino acid level, when compared with the other nineteen DsMV isolates available in GenBank.Among the encoded proteins of three Amorphophallus isolates, the P1 protein was the most divergent of the DsMV isolates, especially P1 of Albus-sym, which had the lowest aa identity (58.96–75.58%) to other DsMV isolates using the BLASTp search, which is less than 82% (potyvirus species demarcation criteria thresh)^16^, CP had the aa identity (87.80–91.90%) to other DsMV isolates, while HC-Pro-P3 -6K1-CI-6K2-VPg-NIa-NIb had the highest aa identity (87.96–92.7%) to other DsMV isolates (Tables S3 and S4). In a phylogenetic tree inferred from the whole genome sequences of DsMV, AK-83, and konjac-sym (both DsMV isolates) formed a distinct clade, along with isolates of DsMV from *Typhonium giganteum* Engl. (Baifuzi) from the Henan Province of China, while an Albus-sym isolate formed a distinct clade with a T10 isolate of *A. paeoniifolius* from India (Fig. S1). These results suggest that Albus-sym is distinct from other DsMV isolates as well as the VanMV strain, whose complete genome is 75.8–77.1% and 83.9–86.7% identical to DsMV at the nt and aa levels, respectively^17^.

To further define the possible mechanisms of DsMV diversity, RNA recombination events between 23 full-length DsMV sequences were examined using the RDP4 software. Only events supported by at least four methods with P-values < 1 × 10^–6^ were considered significant. A total of 51 putative recombination events were detected in 23 DsMV complete genomes (Fig. S2). These recombination events occurred in the entire genome. Of 51 recombination events detected, twelve events were identified from three Amorphophallus isolates of DsMV (Table 1). Four events in Konjac-sym, five events in Albus-sym, three events in AK-83, Konjac-sym and AK-83 shared two same recombination events (events 2 and 10).

**Table 1:** The recombination events identified in three Konjac isolates of DsMV by RDP4 between all available full-length DsMV genomic sequences.

Recombinant	Recombination events	Breakpoint positions^a^		Recombinant sequence			Detection method^d^					
		Begin	End	Minor parental Seq^b^	Major parental Seq^c^	RDP	GENECONV	Bootscan	MaxChi	Chimaera	SiScan	3Seq
Konjac-sym	1	1699	2462	BF1MW701396)	AK-83	4.375E−5	4.589E-17	1.55E−2	6.400E−15	6.877E−9	4.574E−51	NS^d^
	2	3246	4816	Et41(MG602232)	BF1MW701396)	5.394E−14	2.006E−12	1.812E−14	2.563E−10	2.342E−8	1.315E−18	3.377E−4
	3	5336	6777	II(KY242359)	AK-83	1.031E−18	NS	2.920E−14	2.329E−9	1.490E−11	NS	5.129E−13
	4	8981	9117	Et41 (MG602232)	AK-83	2.366E−19	6.929E−14	1.237E−9	5.513E−9	1.913E−9	NS	5.129E−13
Albus-sym	5	696	960	BF1MW701396)	SDP(JX083210)	1.120E7	4.019E−4	8.372E−8	2.811E−4	2.687E−2	1.068E−7	7.430E−4
	6	1148	1383	Et41 (MG602232)	BF1MW701396)	2.554E−11	1.521E−6	3.884E−8	1.837E−5	3.575E−4	4.368E−8	5.131E−8
	7	4300	5749	BF1MW701396)	CTCRI-II-14(KT026108)	6.561E−8	NS	2.964E−7	2.881E−4	2.067E−2	1.984E−6	9.176E−7
	8	6833	8285	CI(KX505964)	UG31 (MG602235)	2.217E−2	NS	NS	3.921E−6	6.313E−8	1.887E−7	1.080E−4
	9	8338	10,131	ET29(MG602230)	T10(KJ786965)	6.254E−20	3.177E−5	1.009E−16	8.935E−16	2.238E−9	2.923E−24	5.656E−4
AK-83	10	1	790	ET26(mg602229)	Et41 (MG602232)	NS	NS	NS	2.7073E−6	6.108E−6	4.288E−14	3.377E−4
	11	3223	4612	ET26(mg602229)	Konjac-sym	3.257E−36	1.529E−25	2.114E−34	1.058E−14	4.541E−17	7.884E−19	5.129E−13
	12	9901	10,098	Et36 (MG602231)	UG31 (MG602235)	3.246E−4	6.960E−12	1.812E−14	2.563E−10	2.342E−8	1.315E−18	3.377E−4

^a^Position refers to the major parental sequence indicated.

^b^Parent strain contributing the smaller fraction of the sequence.

^c^Parent strain contributing the larger fraction of the sequence.

^d^NS: No significant P value was obtained using this method.

We obtained the CP gene sequences of 13 DsMV isolates from 106 diseased *A. konjac* samples that contained DsMV-like particles in TEM analysis with a DsMV 3′-terminal sequence and/or CPF/CPR-specific primer pair (Table S5). Multiple sequence alignment of the CP amino acid sequence of 13 DsMV isolates from this study with that of 26 other DsMV isolates downloaded from GenBank revealed that the N terminal region of DsMV CP is highly variable (Fig. S3), while the C-terminal sequences were highly conserved. Sequence comparisons with the DNAMAN software among the Yunnan isolates showed that these CP sequences were 77.5–100% genetically similar based on nt sequences, and 86.2–100% similar based on aa residues. The Amorphophallus isolates in this study shared the highest nt identity (97.87%) with the JH taro isolate (Acc.no. AFA28134), followed (97.57%) by YN80 isolate^17^ from China. Except for Albus-sym and Konjac-98, the CP size (321aa–329aa) of DsMV Amorphophallus isolates from China was bigger than some isolates such as BF1, BF8, BF39 and _Pinellia_Yunnan isolate. While CPs of Albus-sym and Konjac-98 were the most similar to two konjac isolates (Ds03 and Ds18) from Japan and a Spathiphyllum isolate from India with 79.81–80.44% aa identities. their size (301 aa) were shorter than the other DsMV isolates.

All Yunnan isolates except Albus-sym shared a 10-aa GNNTNTNT(N/S)T sequence repeated three times in tandem (Fig. S3). This sequence repeat has previously been reported in DsMV Nicaraguan isolates and DsMV-LA^18^. Most of the CP of DsMV isolates from taro in Ethiopia, *A. konjac* in Japan, and two isolates from *Zantedeschia aethiopica* in Zhejiang province of China possess a similar N-terminal and conserved motif rich in asparagine residues (Fig. S3). The N-terminal of several isolates such as isolates CI (CAE83573) from Vallin, SY1(CAF32246), Pinellia tuberifera tenore (ACA28634) from Pinellia ternate in China, Ds11(BAU36925) and Ds12 (BAU36926) from Japan, and Albus-sym, all possessed a proline-rich motif with 5 or 6- proline residues similar to all the DsMV isolates.

**Figure 4:**
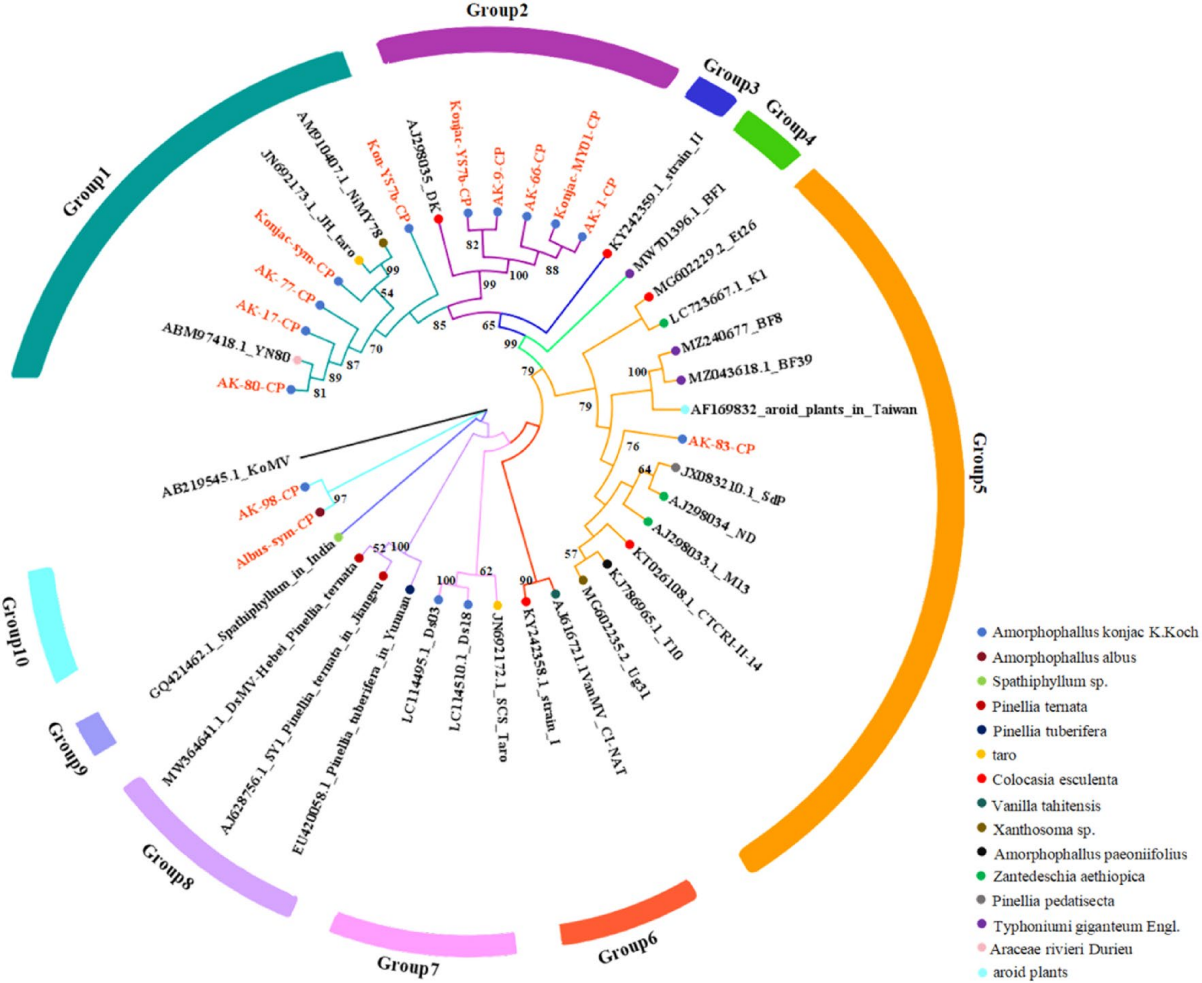
Phylogenetic tree based on CP nucleotide sequences of 13 dasheen mosaic virus (DsMV) isolates (this study) and 26 DsMV isolates (NCBI). The tree was constructed in MEGA-X using the maximum likelihood method and General Time Reversible model 111 with 1000 bootstrap replications. Bootstrap values less than 50 are not shown. Bars and branches with different colors denote ten groups of DsMV, respectively. Circles with different colors represent different hosts of DsMV.

The aa triplet DAG, related to aphid transmission^19^, was present in most of the Yunnan DsMV isolate sequences, except for the Albus-sym isolate and AK-98, the DsMV-Amp6 isolate (ADN88392.2) from *A. paeoniifolius* in India, and the isolate BAU36932 from konjac in Japan, where the DAG was changed to NAG (Fig. S3). Phylogenetic relationships based on aa sequences of the CP gene indicated that all the DsMV isolates were grouped into ten distinct clades based on geographic location and host plant species where the samples from Yunnan were divided into five groups (Fig. 4).

The RNA extracts of All 106 samples with DsMV-like particles tested for the presence of DsMV by RT-PCR using the degenerated primers, CIFor/CIRev^20^, showed the expected 683 bp amplicon in 48 *A. konjac* samples, 13 *A. albus* samples, and 7 *A. bulbifer* samples. A total of 30 partial CI gene sequences were obtained including 16 from diseased *A. konjac* samples, 13 from diseased *A. albus* samples, and one from buckwheat showing chlorotic spot symptoms. Alignment showed these sequences had 75.5–97.7% nt identities and were divided into six clusters (nt identity less than 86%). The partial CI gene sequence of Albus-sym shared 93–97% nt identity with isolates from *A. konjac* or *A. albus*. On the contrary, the partial CI gene sequence of Konjac-sym shared relatively low nt identities (less than 85%) with other DsMV isolates from Yunnan.

**Figure 5:**
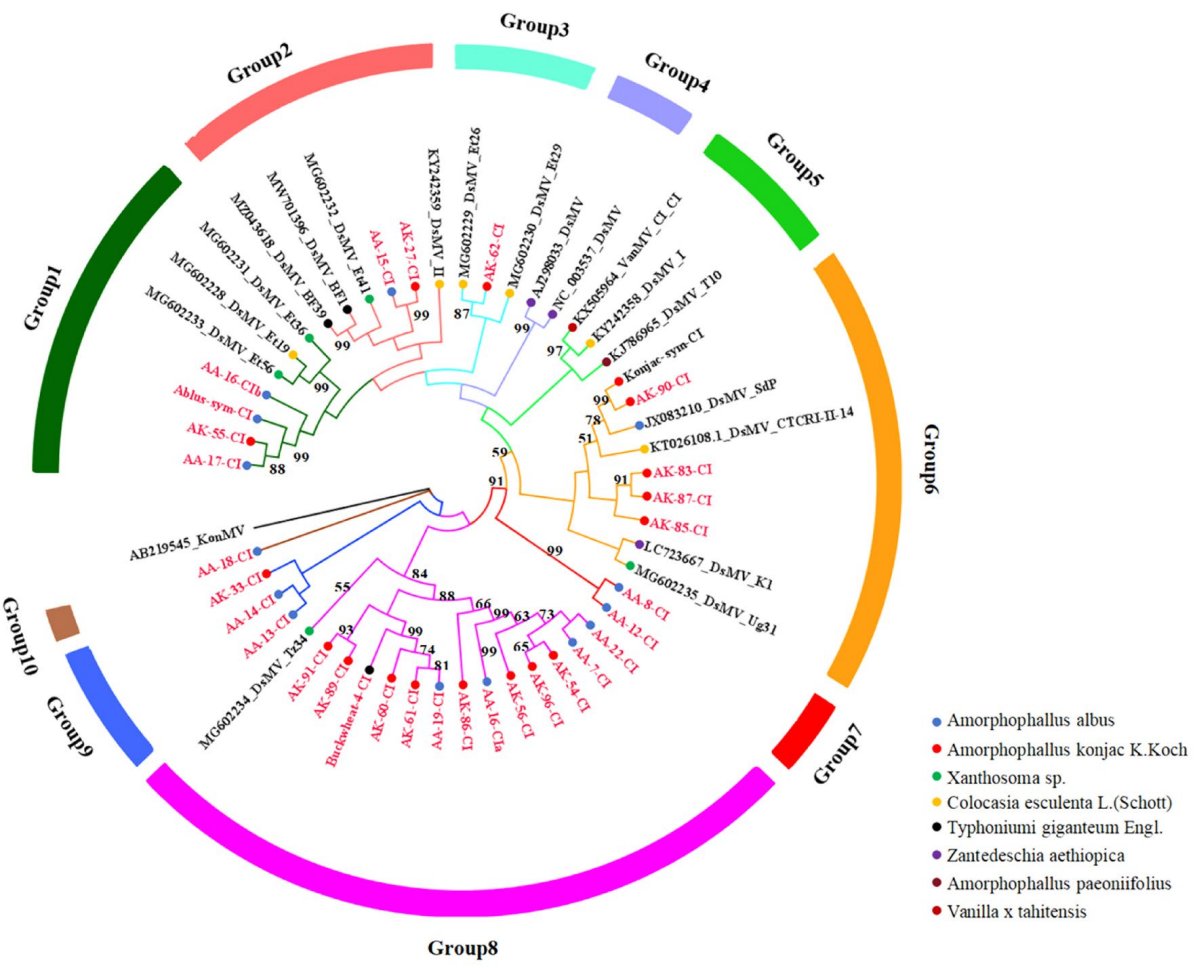
Phylogenetic tree based on the core nucleotide sequences of CI proteins of 31 dasheen mosaic virus (DsMV) isolates (this study) and 19 DsMV isolates (NCBI). The tree was constructed in MEGA 11 using the maximum likelihood method and General Time Reversible model 111 with 1000 bootstrap replications. Bootstrap values less than 50 are not shown. Bars and branches with different colors denote ten groups of DsMV, respectively. Circles with different colors represent different hosts of DsMV.

Phylogenetic analysis results using the amino acid sequences of the CI gene from Yunnan DsMV isolates, together with corresponding sequences of published DsMV and konjac mosaic virus as an outgroup are shown in Fig. 5. The DsMV isolates grouped into ten distinct clades based on geographic location and host plant species. DsMV isolates from Yunnan were divided into eight groups.

Results based on the sequence analysis of CP and CI sequences also showed that Konjac-sym is the dominant isolate of DSMV infecting konjac in Fuyuan County of Yunnan, which is a seed corm production plantation.

### Three orthotospoviruses infecting *Amorphophallus*

BLASTx analysis of the assembled contigs from the four HTS-sequenced samples revealed that 11 long contigs (1915–8968 nt in length) from Albus-sym had the highest nucleotide similarities to MVBaV and TZSV. These contigs mapped to the Nucleoprotein, Non-structural protein (NSs), Movement protein, Glycoprotein and RdRp of MVBaV and the Nucleoprotein, Non-structural protein (NSs), Glycoprotein and RdRp of TZSV.

The RNA segments of the MVBaV Konjac isolate were determined to be 8909 nt (L, accession no.OR311349), 4707 nt (M, OR311348), and 2948 nt (S, OR311347) in length by RT-PCR. They shared a sequence identity of more than 84% with an isolate YN-Pharbitis purpurea from Kunming in Yunnan (segment L, 96.18%, MK681486.1), segment M, MK239333.1, 86.99%) and isolate NN-16 from mulberry in Nanning (segment S, KM819708.1, 84.07%).

The RNA segments of the TZSV Konjac isolate genome were determined to be 8918 nt (L, OR311352), 4758 nt (M, OR311351), and 3336 nt (S, OR311350) in length by RT-PCR. They shared a sequence identity of more than 95% with isolates of KM-tomato from Kunming, Yunnan, China (segment L, 95.96%, EF552435 and (segment M, EF552434, 97.91%) and isolate TZSV-WS1901 (segment S, 97.37%) from pod pepper in China.

The nucleocapsid (N) gene (OR911346) of the INSV Konjac isolate were obtained by RT-PCR with the specific primer pair (INSV-NF/R) (Table S2). It shared an identity of 99.87% with INSV TV isolate from *Tulbaghia violacea*.

**Figure 6:**
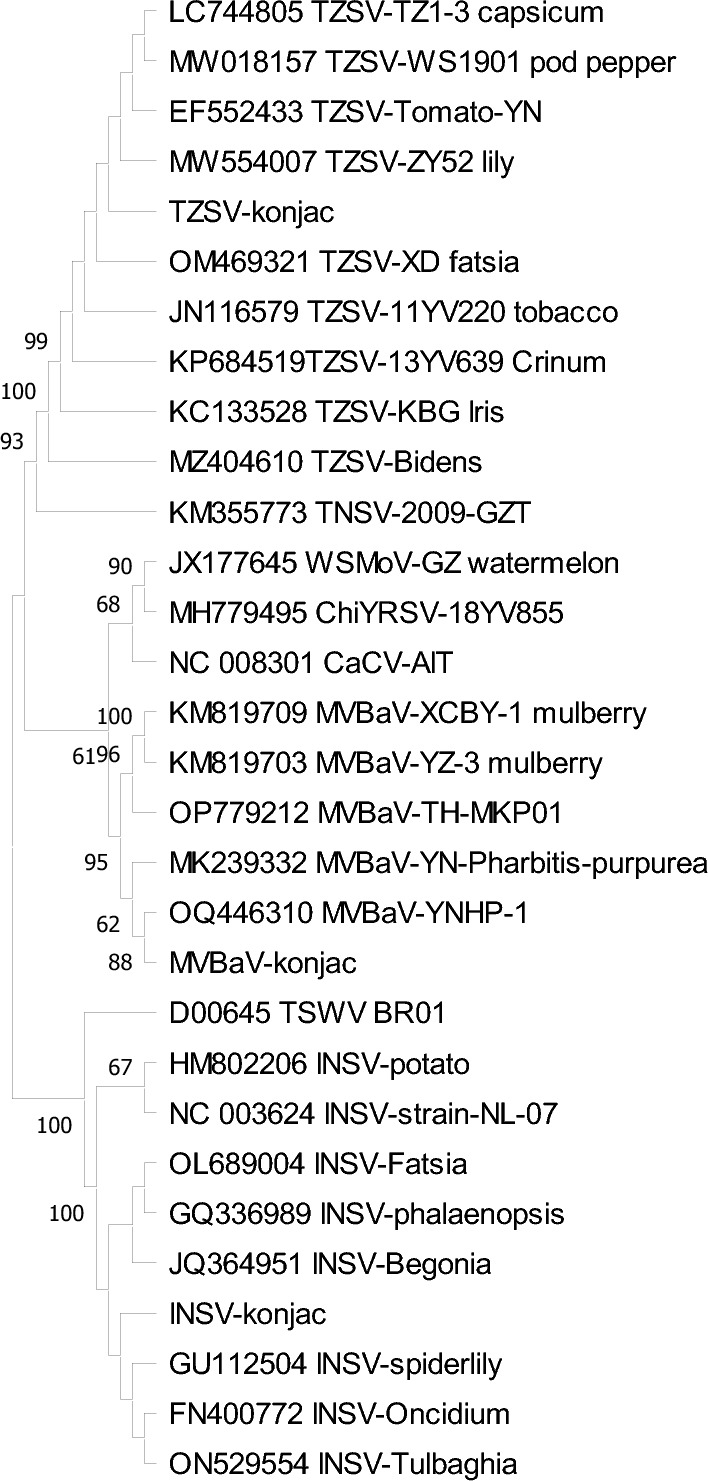
Phylogenetic trees of orthotospoviruses based on the amino acid sequences of the N protein of three orthotospoviruses in this study and other orthotospovirus in the GenBank. Phylogenetic trees were generated according to the Maximum Likelihood method and Tamura-Nei model in MEGA11. Bootstrap values on the branches represent the percentage of 1000 bootstrap replicates. Bootstrap values greater than 50% are shown. Virus abbreviations: TZSV, tomato zonate spot virus; TNSV, tomato necrotic spot associated virus; ChiYRSV, chili yellow ringspot virus; WSMoV, watermelon silver mottle virus; CaCV, capsicum chlorosis virus; MVBaV, mulberry vein banding-associated virus; TSWV, tomato spotted wilt virus; INSV, impatiens necrotic spot virus.

In a Maximum Likelihood phylogenetic tree based on nucleocapsid aa sequences of orthotospoviruses, three orthotospovirus isolates from Amorphophallus clustered with a subclade of MVBaV, TZSV and INSV isolates, and appeared most closely related to those isolates from Yunnan province (Fig. 6).

**Figure 7:**
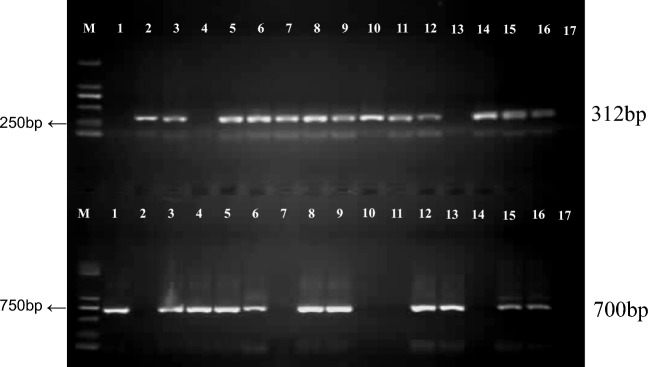
RT-PCR detection of mixed infection in diseased Amorphophallus samples. M represents DL2000 DNA Marker (TaKaRa, Beijing, China). (**A**) Detection of orthotospoviruses with dTosp-F2 and dTosp-R2, the length of amplicon is 312 bp; (**B**) detection of DsMV with CIFor/CIRev, the length of amplicon is 683 bp. Lane 1–6: Konjac-47, 49–53; Lane 7–8: Albus-15, 16; Lane 9: Albus-18; Lane10–11: Konjac-53, 54; Lane12: Kojac-59; Lane 13: Albus-19; Lane 14: Albus-20; Lane 15: Konjac-60; Lane 16: Konjac-62; Lane 17: Mock.

RT-PCR detection with the degenerated primer pair (dTospo-F2 and dTospo-R2)^21^ and Sanger Sequencing also confirmed infection of the two orthotospoviruses to Amorphophallus seedlings (Fig. 7). Among the randomly selected 17 samples, 13 sampled were infected by DsMV,11 samples were infected by orthotospovirus, 8 samples were coinfected by DsMV and orthotospoviruses.

### RT-PCR detection of orthotospoviruses infecting *Amorphophallus*

The RT-PCR detection of orthotospoviruses with the degenerated primers dTospo-F2/dTospo-R2^21^ revealed that 27 (45.7%), among 59 Amorphophallus samples subjected to RT-PCR assays, were positive. In addition to MVBaV and TZSV, INSV was found from two samples of *A. bulbifer*. The obtained N gene and partial sequence of the INSV L RNA shared a nucleotide sequence identity of more than 98.6% with the INSV TV isolate (ON529552) from *Tulbaghia* violacia Harv.

### DsMV and orthotospoviruses mixed infection in *Amorphophallus*

The RT-PCR analysis using the degenerate primers CIFor/CIRev for potyviruses and dTospo-F2/dTospo-R2 for orthotospoviruses combined with negative staining electron microscopy showed that DsMV, MVBaV, TZSV and INSV occurred in both single and mixed infections in *Amorphophallus* (Fig. 7 and Table S1). We detected DsMV in 125 of 146 (86.2%) of the *Amorphophallus* samples from Yunnan province, China. Similarly, MVBaV was detected from 16 *A. konjac* samples, two *A. bulbifer* samples from Kunming and five *A. albus* samples, while TZSV was detected from six *A. konjac* samples from Fuyuan County, one *A. bulbifer* sample from Kunming, and four *A. albus* samples. INSV was detected from one *A. konjac* sample and one *A. bulbifer*. Based on electron microscopy and RT-PCR, mixed infections of Amorphophallus were found in 79 of the 122 samples. A total of 16 samples were co-infected by DsMV and MVBaV. One *A. bulbifer* sample (Bulbifer-7) was co-infected by the above mentioned four viruses.

### No viruses were detected from seedlings propagated from seeds

The seedlings propagated from seeds derived from symptomatic and asymptomatic *A. konjac* plants and F1 seeds from hybrids between *A. konjac* and *A. albus* were screened for viral presence using RT-PCR with the degenerated primers CIFor/CIRev and dTospo-F2/dTospo-R2 for potyviruses and orthotospoviruses, respectively, and no amplicons of the expected size were obtained from these seedlings (n1, n2 = 30).

### Intermediate hosts of viruses infecting *Amorphophallus*

**Figure 8:**
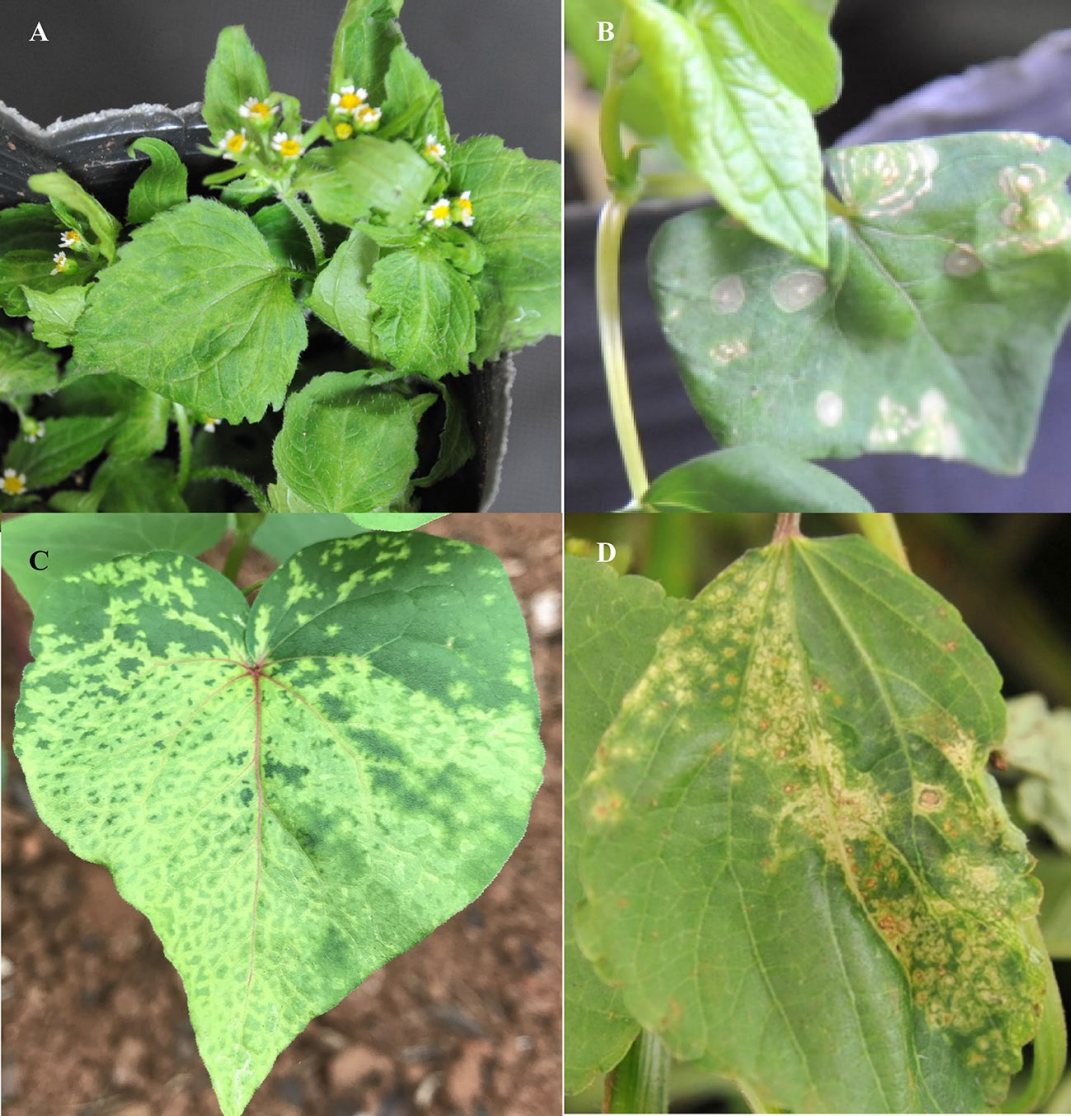
Natural infection of DsMV and MVBaV in the weeds, *Galinsoga qurdriradiata* (**A**, Galinsoga-3), *Fagopyrum esculentum* Moench (**B**, Buckwheat-3; **C**, Buckwheat-4) and *Acalypha australis* (**D**, Acalypha-1) in the konjac growing fields. DsMV and MVBaV were detected from (**A**–**C**). MVBaV from (**D)**.

To determine the intermediate hosts of viruses infecting Amorphophallus, we performed a field survey and fully observed the weeds and other crops exhibiting obvious virus symptoms (Fig. 8) in/around the Amorphophallus fields in Fuyuan County during the growing seasons (from July to August). 15 symptomatic weeds and crops were collected for RT-PCR and EM testing. The testing results showed that DsMV were present in the leaves of *Galinsoga quadriadiata*, MVBaV in *Acalypha australis*, *Fagopyrum esculentum* Moench, and chili pepper (*Capsicum annuum*), TZSV in *Hippeastrum rutilum* (Ker-Gawl.) Herb, and iris, INSV in Tulbaghia violacea Harv., *Frankliniella occidentalis* (Pergande) (Thysanoptera: Thripidae) accounted for approximately 90% of the thrips captured in aerial traps.

## Discussion

Incidences of DsMV causing mosaic symptoms in *A. konjac* have been reported in Yunnan, China^11^. In recent years, many new symptoms, including leaf mosaic, mottling, chlorotic spotting, ringspot, puckering, leaf deformation, shoestringing, feathery mottle, and stunt have been described in *Amorphophallus* plants^6^. The incidences of mild mottle and chlorotic symptoms, which are usually considered physiological diseases by some growers and agricultural technicians, reached more than 50% in some fields. It is concerning that the ignorance and unclear understanding of the presence of viral diseases in *Amorphophallus* species could lead to the spread of the virus through seed corm. Thus, the pathogens underlying these symptoms need to be systematically identified.

Using a combined approach with HTS-based sequencing, RT-PCR, electron microscopy, and Koch’s Postulate verification, several known viruses (DsMV, MVBaV, TZSV, and INSV)were detected and identified from 146 collected *Amorphophallus* samples. Among these, DsMV is the dominant viral pathogen in *Amorphophallus*, causing mosaic, mottle, chlorosis, and stunt symptoms. We picked up on frequent mixed infections in the fields, and mixed infections of DsMV and MVBaV were detected in 16 samples through the RT-PCR.

Sequence analysis based on the CP and CI sequences showed that the DsMV *Amorphophallus* isolates in Yunnan have abundant genetic diversity. Recombination analysis with the RDP4 software based on the nucleotide sequences of the complete genome indicated that recombination in the HC-Pro, P3, NIa, NIb, and CP gene regions could be important drivers in DsMV diversity. Previous studies have also reported high levels of genetic variation in the genome of DsMV^22,23^. For example, the CP gene sizes exhibit variation in size, ranging between 936 and 1008 nt in DsMV *Amorphophallus* isolates, primarily due to variations in the 5′ region of the CP genes (312 aa–335 aa) stemming from deletions or insertions sequence of CP genes. A particular characteristic of the DsMV *Amorphophallus* isolates is that these isolates (except for isolate Albus-sym) shared a 10-aa GNNTNTNT(N/S) T sequence repeated three times in tandem. These results suggest that DsMV isolates are genetically diverse, as suggested by Li et al.^24^. However, it is currently unclear whether there is a direct correlation between this repetition and the origin, host, or geographical location of the DsMV strain.

DsMV is a positive sense, single-strand RNA virus that belongs to the genus *Potyvirus* of the family *Potyviridae* with a typical host range including Araceae taro, Konjac, cocoyam, caladium and calla lilies worldwide^25–28^. In China, several reports have published the complete sequences of DsMV isolates, for example, the M13 isolate from Zantedeschia aethiopica (NC_003537.1)^22^, the BF1 and BF39 isolates from *Typhonium giganteum*^14^, the SdP isolate (JX083210.1) from Pinellia pedatisecta, and the SY1 isolate (AJ628756) from *P. ternata*^29^. Although we have reported the sequence of the 3′-end of DsMV Konjac isolate from *A. konjac* in Qujing (Yunnan province), there still was no report of the complete sequence of a DsMV isolate from *Amorphophallus* in China^11^. Thus, here we report on the first complete sequence of three DsMV isolates from *A. konjac* and *A. albus*, where our results indicated that the genetic variation in DsMV infecting *Amorphophallus* could depend on the host.

Orthotospoviruses are enveloped plant viruses with a three-segmented, linear, negative-sense single RNA strand. They cause significant losses in vegetables and high-value crops worldwide^30^. Thus, an increasing number of orthotospoviruses, including MVBaV, TZSV, and INSV have been found in agricultural crops such as mulberry^31^, tomato^32^, tobacco^33^, chili pepper^34^, ornamental plants such as iris^35^, Crinum asiaticum^36^, spider lily^37^, and Tulbaghia violacea Harv^38^, and weeds such as Pharbitis purpurea^39^ and bidens^40^ in many regions of China^41^. Previously, tomato spotted wilt virus (the typical virus in the genus *Orthotospovirus*) was suspected of infecting konjac based on the morphology of the virus particles based on the electron microscopy^41^. The multi-prong approach taken by our study confirmed the presence of three orthotospoviruses including MVBaV, TZSV, and INSV in *Amorphophallus* in China using molecular and etiological evidences to confirm the infection based on the molecular characterization and Koch’s Postulates.

Infection of *A. konjac* by konjac mosaic virus (KMV) and cucumber mosaic virus (CMV) have been reported previously^7,12^. However, we did not detect these two viruses using the specific primer pairs (Table S2) for the CP genes^42^ from the *Amorphophallus* samples collected from the 2018–2019 growing seasons (data not shown).

Edible Amorphophallus plants such as *A. konjac*, *A. albus*, and A. bulbifer play a very important role in the socio-economic and agricultural scene of China^3,43^. Since these corm crops usually use the seed corm to vegetatively propagate seedlings in agricultural production, they indefinitely pass the virus to the new seedling. Due to the relatively mild symptoms of this viral disease, in contrast to bacterial soft rot, Amorphophallus diseases are often ignored in production, and since the viral infection spreads by seed corms and results in the reduction of corm yields, the industry is in desperate need of effective management strategies for controlling virus diseases. In the absence of an agrochemical that specifically targets plant viral infection cycles, cultivating varieties with resistance to either viruses or their vectors is one of the most economical and effective way to control viral diseases^44,45^. Under the circumstances, with a lack of DsMV, orthotospoviruses, and vector resistant varieties of Amorphophallus, more research is necessary on the molecular characterization of Amorphophallus-infecting viruses including the comparative analysis of viral genomes, and analysis of genetic variation between different isolates^30,46^. These studies would facilitate the development of diagnostic tools to monitor the viral spread and identify seed corms for quarantine, screen viruses for plant tissue culture, investigate the virus-plant pathosystem, and ultimately enable the development of resistant varieties.

## Conclusion

This is the first comprehensive report on DsMV and orthotospoviruses infecting Amorphophallus plants in Yunnan province, China. These findings will aid agricultural researchers and farmers in this region to develop integrated management strategies including the development of virus-free seed corms through meristematic tissue culture, the selection and breeding of virus resistance germplasms, the timely removal of infected intermediate hosts including *F. esculentum*, *G. quadriadiata*, *A. australis*, *Hippeastrum sp*. and *Capsicum annuum*, and the bio-control of virus vector populations. Ultimately, in conjunction with future studies, this work will help to sustain the crop-based economy of Amorphophallus growing regions.

## Methods

### Sample collection and field survey

Plants and tissues were mainly collected from plants displaying severe symptoms, such as black necrosis and concentric ringspot symptoms on leaves during the 2016–2021 growing seasons. Entire plants of *A. konjac* and *A. albus* were collected from six sites in Fucun town, Laochan town, Zhuyuan town, Zhongan town, Mohong town, Housuo town from Fuyuan County, one site in Baishui from Zhanyi County, one site in Laowo from Lushui City, and one site in Beidou from Yongping County. Entire plants of *A. bulbifer* were collected from two sites in Fuyuan County and one site in Kunming City. Additionally, the disease incidence in the field was estimated. The viral transmission ratio of *Amorphophallus* seed corm has to be understood from a large-scale field survey over four years (from 2019 to 2022). Thrips in the field were trapped by blue plate and identified by morphology. Additionally, a total of 160 samples, including 146 Amorphophallus samples and 14 samples of weeds and crops, showed virus-like or unusual symptoms and were collected from six counties in Yunnan province. The Amorphophallus samples’ symptoms included leaf mosaic, mottling, chlorotic spotting, puckering, leaf deformation, feathery mottle, and stunted growth. The entire plants of partial samples were uprooted from field and transplanted into the greenhouse of Fuyuan Konjac Institute located in Fuyuan County and the Yunnan University of Chinese Medicine for later sampling and testing. Part of the diseased leaves were stored at – 80 ℃. Samples were labeled with *Amorphophallus* sp., description of leaf symptoms, and collection location (Listed in supplementary Table S1).

### Plant inoculation

All plant seedlings including A. konjac, *A. albus* and other non-Amorphophallus plants such as *C. amaranticolor, N. benthamiana, B. Pilosa,* buckwheat (*F.* *esculentum*) were obtained through seed germination and were grown in a growth chamber set to 25/18 ℃ (day/night) with a 16/8 h light/dark photoperiod. Leaves of Amorphophallus samples (Konjac-47, konjac-51 and konjac-56) were ground, respectively, with a mortar and pestle in phosphate buffer (0.033 M KH_2_PO_4_, 0.067 M K_2_HPO_4_ and 0.01 M Na_2_SO_3_, pH 7.0)^47^. The resulting saps were used as the inoculum to mechanically inoculate the above-mentioned seedlings. Two of the initial four leaves of plant seedlings were mechanically inoculated. The single virus isolate of DsMV (Konjac-51), MVBaV (Konjac-47) and TZSV (Konjac-56) were obtained, respectively, through two passages of single local lesions in *C. amaranticolor*. The single-lesion isolate of DsMV was maintained on *A. konjac*, MVBaV and TZSV on *N. benthamiana*. . For mock-inoculated control plants, leaves were rub-inoculated with the phosphate buffer alone. Three biological replicates were performed, where every replicate included six plants. The symptomatic and mock-inoculated leaves were then collected at 14 dpi and stored at – 80 ℃ for RT-PCR assay.

### Electron microscopy observation

A pioloform carbon-coated copper grid was floated for 3.5 min on the crude sap extracted from diseased leaf tissues to absorb the viral particles. The grids were then transferred onto one drop of 20 µL negative staining buffer (2% ammonium molybdate at pH 6.5) and stained for 2 min. The virions were examined under a transmission electron microscope (FEI TECNAI G2, ThermoFisher Scientific, Hillsboro, Oregon, USA). Ultrathin sections of leaf tissues of 11 Amorphophallus samples were prepared according to Dong et al.^32^. and were examined with the same electron microscope.

### High-throughput sequencing and sequence assembly

Total RNA was extracted from four samples: Konjac-sym, a mosaic and necrotic leaf of *A. konjac*, Konjac-asym, an asymptomatic leaf of an *A. konjac* seedling regenerated through meristem tissue culture, Albus-sym, a mosaic leaf of *A. albus*, and Albus-asym, an asymptomatic leaf of an *A. albus* seedling regenerated through meristem tissue culture, using TRIzol (Ambion, Hillsboro, Oregon, USA) following the manufacturer’s protocol. Ribosomal RNA was depleted using a RiboZero kit (Illumina, San Diego, CA, USA) according to the manufacturer’s protocol. Next, four cDNA libraries were synthesized using the TruSeq Stranded Total RNA with Ribo − Zero Gold (Illumina). The cDNA libraries obtained were subjected to deep sequencing using the Illumina HiSeq 2500. The transcriptome sequencing and analysis were conducted by OE Biotech Co., Ltd. (Shanghai, China).

Raw data (raw reads) was processed using Trimmomatic (0.36)^48^. Low-quality reads were removed. Next, the clean reads were de novo assembled into transcripts by using Trinity (version 2.4) with the paired-end method^49^. The longest transcript was chosen for subsequent analysis as a unigene based on similarity and length. The assembled contigs (above 150 nt) were subjected to local BLAST searches against the reference viral sequence (RefSeq) database of NCBI (the E − value cut − off was > 0.05 for local Blastx) (ftp://ftp.ncbi.nih.gov/genomes/Viruses/).

### RNA extraction, RT-PCR amplification, and sequencing

To determine the genome sequence or validate the annotated virus fragments, specific primers (Table S5) were designed based on the contig sequences obtained in this study and were then used in RT-PCR for the amplification of overlapped viral fragments. Total RNA was extracted from fresh leaves using 1 mL TRIzol™ Plus RNA Purification Kit (Thermo Fisher Scientific, Waltham, MA, USA), and 0.2 mL chloroform, precipitated by the addition of 0.5 mL isopropanol, and then washed with 75% ethanol and air-dried. The RNA pellet was dissolved in RNase-free water and analyzed by gel electrophoresis on 1% agarose gels. Total RNA was used to perform one-step RT-PCR amplification using the TaKaRa one-step RNA PCR Kit (TaKaRa, Beijing, China) according to the manufacturer’s instructions. The primer pairs used for RT-PCR are listed in Supplementary Table S2. The expected RT-PCR products were cloned into a pMD18 T easy vector and three independent clones were bidirectionally sequenced by Sanger dideoxy sequencing at the Kunming Branch of Beijing Tsingke Biotech Co., Ltd. (Kunming, China).

To complete the full genome of DsMV, RT-PCR was performed using four overlapping fragments with the primer pairs detailed in Qin et al.^14^. The 3′- untranslated region (UTR) sequences were obtained from PCR amplifications reported by Chen et al. coupled with a primer designed from the 3′-terminus of a specific DsMV sequence (Acc. No. AJ298033)^15^.

To complete the full-length genome of the TZSV and MVBaV Konjac isolates, RT-PCR was performed with four overlapping primer pairs designed from contig sequences (Supplementary Table S2). These amplicons were then cloned and Sanger sequenced. The resulting sequences were assembled and analyzed using DNAMAN version 7.0 (Lynnon Biosoft, Vaudreuil, QC, Canada).

### Sequence analysis

Sequences were compared with those of published viruses in NCBI database using DNAMAN7.0 software (LynnonBiosoft, USA). The nucleotide sequence identity values were obtained in Tables S4, S5. Open reading frames (ORFs) were predicted using the ORF Finder program of the NCBI website (https://www.ncbi.nlm.nih.gov/orffinder/). Phylogenetic relationships between the virus isolates in this study and other published DsMV isolates and orthotospoviruses in GenBank were analyzed in Mega 11 using nucleotide or amino acid sequences of entire or partial proteins^50^. Phylogenetic trees were constructed using the Maximum Likelihood algorithm and General Time Reversible model 111 with 1000 bootstrap replicates. Recombination analyses were performed on the complete genome nucleotide alignment of 23 DsMV isolates in the Recombination Detection Program version 4 (RDP4). Seven methods, including RDP, GENECONV, Chimaera, MaxChi, BootScan, SiScan, and 3Seq were used to detect recombination events and recombination breakpoints using default settings^23,51^. Recombination events were noted if supported by at least three different methods (P-values < 1.0 × 10^−6^).

### Supplementary Information

Supplementary Figures.

Supplementary Figure S2.

Supplementary Table S1.

Supplementary Table S2.

Supplementary Table S3.

Supplementary Table S4.

## Data Availability

The datasets generated during and/or analyzed during the current study are available from the corresponding author on reasonable request.
