# Supplementary material for: Occurrence and characterization of viruses infecting Amorphophallus in Yunnan, China
Source: Sci Rep. 2024 Jun 5;14:12948. doi: 10.1038/s41598-024-63477-y (PMC11153213; doi:10.1038/s41598-024-63477-y)
Supplement: Supplementary file 1 — Supplementary Figures. [file 41598_2024_63477_MOESM1_ESM.pptx]

## Slide 1
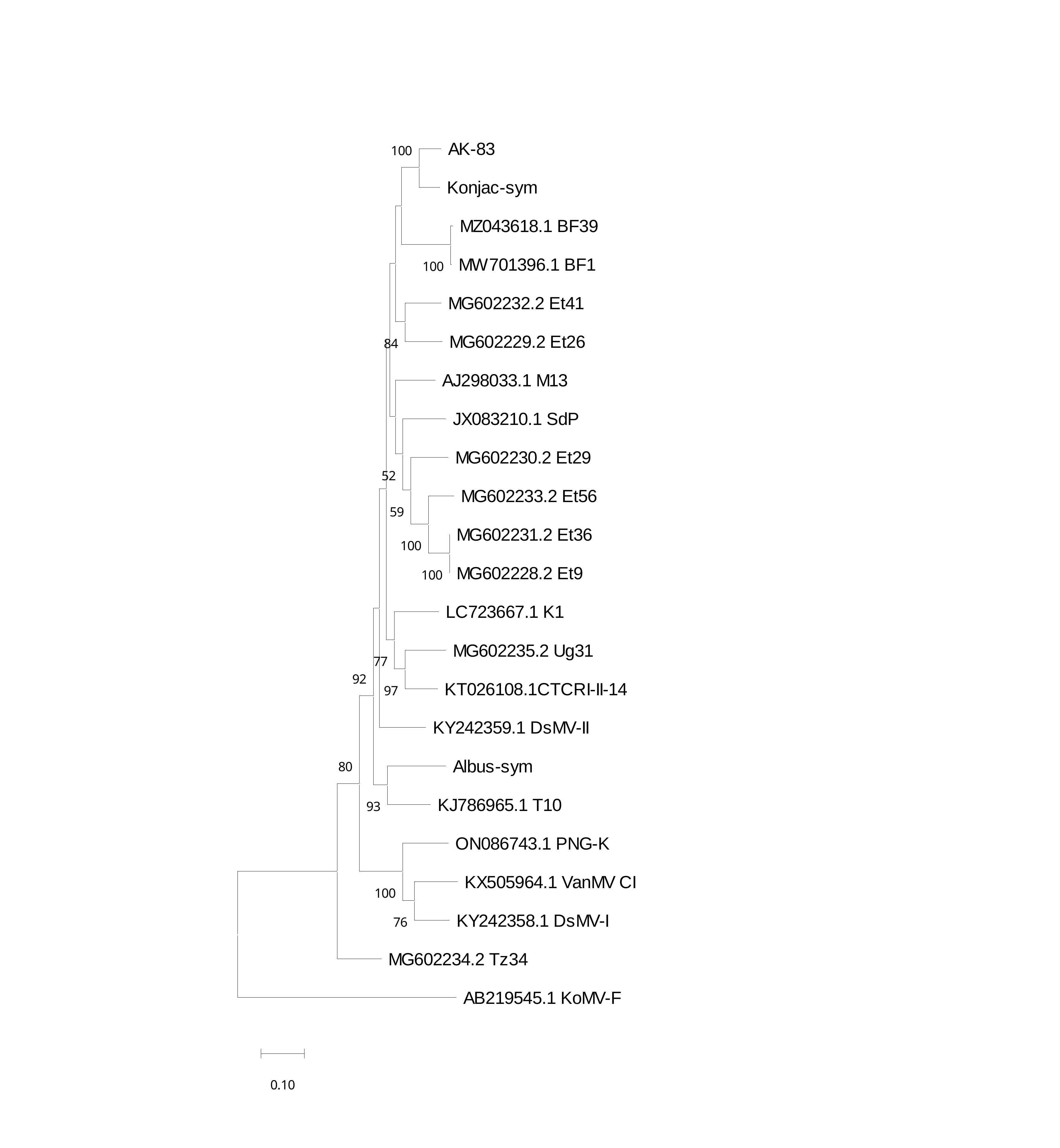

## Slide 2
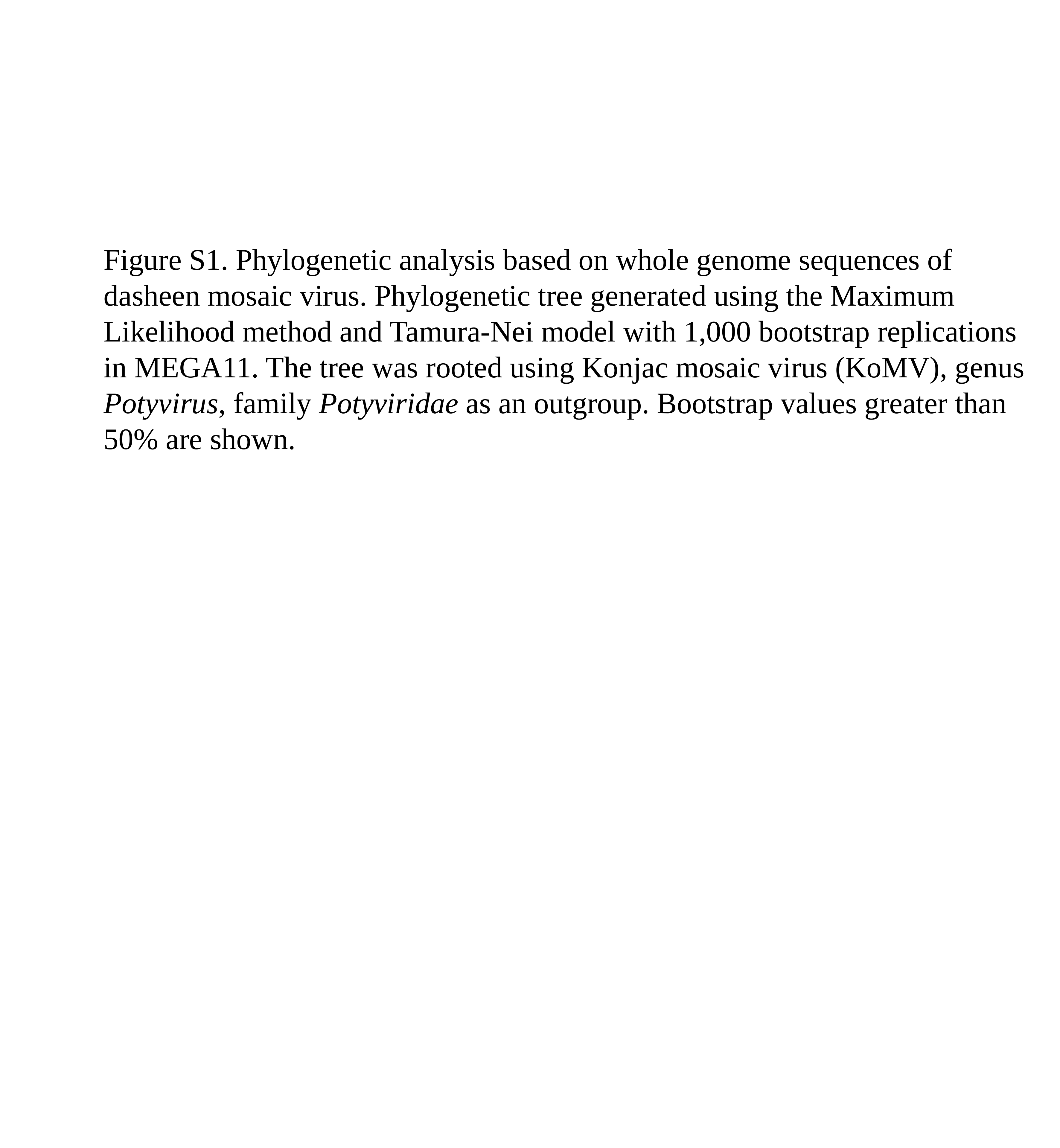

Figure S1. Phylogenetic analysis based on whole genome sequences of dasheen mosaic virus. Phylogenetic tree generated using the Maximum Likelihood method and Tamura-Nei model with 1,000 bootstrap replications in MEGA11. The tree was rooted using Konjac mosaic virus (KoMV), genus Potyvirus, family Potyviridae as an outgroup. Bootstrap values greater than 50% are shown.

## Slide 3
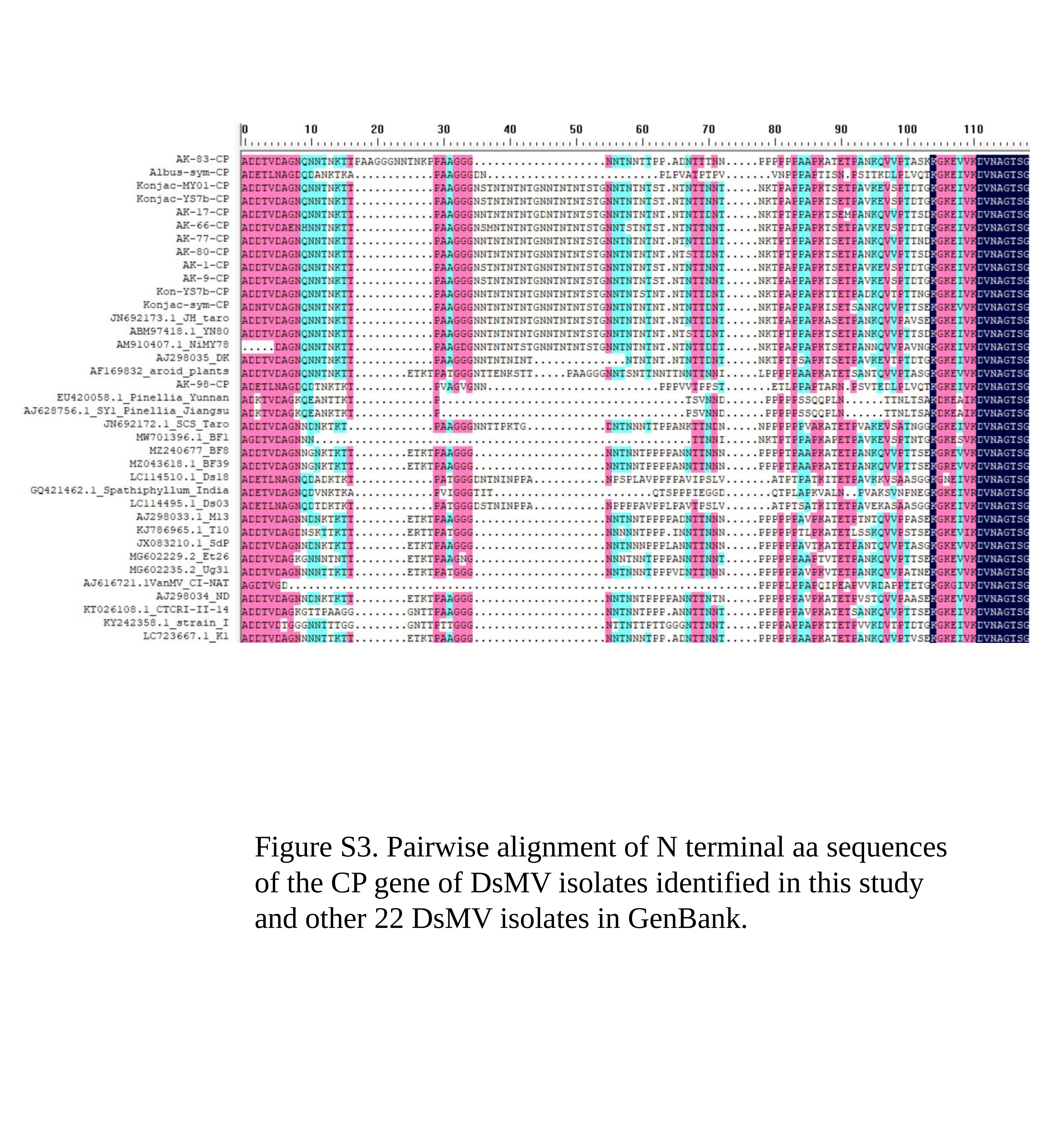

Figure S3. Pairwise alignment of N terminal aa sequences of the CP gene of DsMV isolates identified in this study and other 22 DsMV isolates in GenBank.
