# Supplementary material for: Occurrence and characterization of viruses infecting Amorphophallus in Yunnan, China
Source: Sci Rep. 2024 Jun 5;14:12948. doi: 10.1038/s41598-024-63477-y (PMC11153213; doi:10.1038/s41598-024-63477-y)
Supplement: Supplementary file 2 — Supplementary Figure S2. [file 41598_2024_63477_MOESM2_ESM.docx]

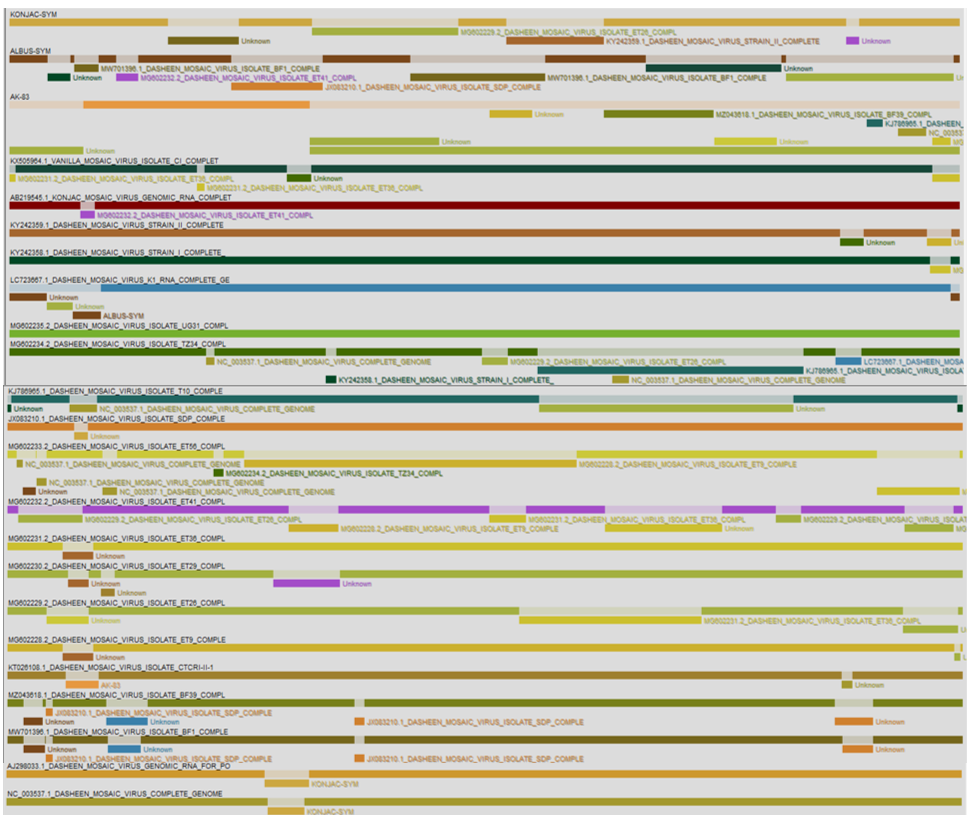


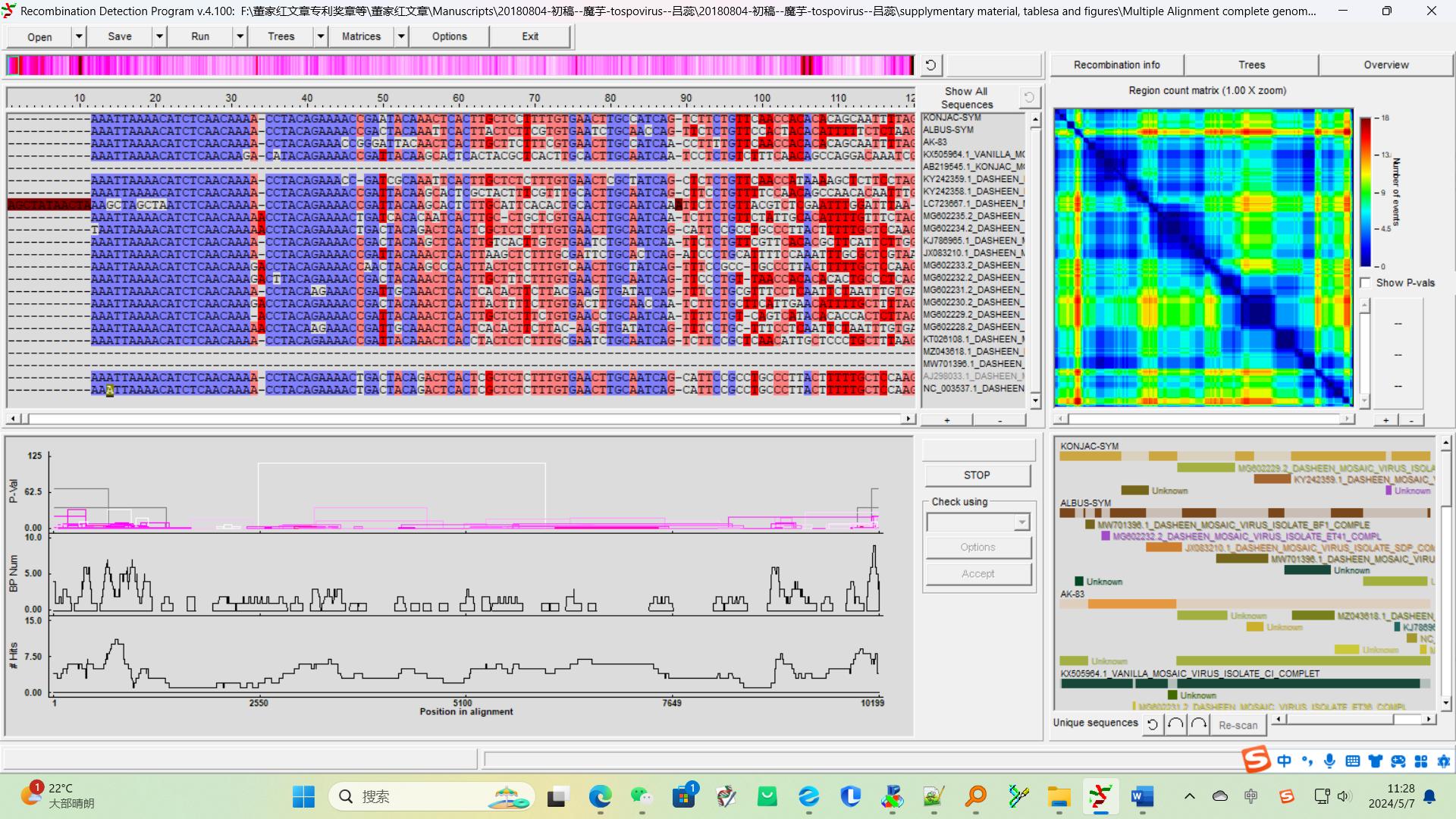


Figure S2. The putative recombination events in Genome RNA of 23 DsMV isolates using RDP4. First panel: Possible recombination events are indicated by short bars. Second panel: The distribution of hotspot sites in the DsMV genome. P-Val: the minimum probability values associated with detected events; BP Num: Breakpoint number; #Hits: the number of events detected in particular regions of the alignment.
