# Supplementary material for: Occurrence and characterization of viruses infecting Amorphophallus in Yunnan, China
Source: Sci Rep. 2024 Jun 5;14:12948. doi: 10.1038/s41598-024-63477-y (PMC11153213; doi:10.1038/s41598-024-63477-y)
Supplement: Supplementary file 6 — Supplementary Table S4. [file 41598_2024_63477_MOESM6_ESM.docx]

Table S5 Primers used for virus detection and genome sequence amplification with RT-PCR

| Primer name | Primer sequence (5’-3’ ) | Expected size (bp) | Target region | Reference/notes |
| --- | --- | --- | --- | --- |
| CIRev | GGIVVIGTIGGIWSIGGIAARTCIAC | 700 | Cylindrical inclusion protein | Ha et al. (2008) |
| CIFor | ACICCRTTYTCDATDATRTTIGTIGC |  |  |  |
| CMV-CPF | ATGGACAAATCTGAATCAACCAGTG | 660 | Coat protein | Wang et al. (2019) |
| CMV-CPR | TCAAACTGGGAGCACCCCAGATG |  |  |  |
| KoMV-CP-F | GGGATAAGAAGTTGGATGC | 700 | Coat protein | Designed from the KoMV (AB219545) |
| KoMV-CP-R | TGTCTCTCTGTGTCCTCTTCC |  |  |  |
| dTospo-F2 | GATCAATCNAARTGGTCDGCWTC | 312 | consensus sequences of orthotospoviral L RNAs | Huang et al. 2018 |
| dTospo-R2 | CATDGCACAAGARTGRTAVACWGA |  |  |  |
| U341 | CCGGAATTCATGRTITGGTGYATIGAIAAYGG | 900 | 5’-terminal of CP gene, Primers used to validate DsMV isolates | Yamamoto and Fuji (2008) |
| Sprimer | GGAAYAAYAGYGGCARCC | 1600-1900 | 3’-terminal of potyviruses genome | Chen et al.2002 |
| M4-T | GTTTTCCCAGTCACGACTTTTTTTTTTTTTTT |  |  |  |
| M4 | GTTTTCCCAGTCACGAC |  |  |  |
| primers for genome of DsMV | | | | |
| DSMV-1F | AAATTAAAACATCTCAACAAAACCTACAG | 780 | 5’-terminal of potyviruses genome | Qin et al, 2021 |
| DsMV-780R | TAGTGTGTTGCAGGGAGCGCAATAA |  |  |  |
| DsMV-721F | TTGTGGTTGCTAATGCGCATAAGGT | 2200 |  | Designed from the contigs |
| DsMV-2940R | TTTCAGTAAGGGCCTTGTTGGACAT |  |  |  |
| DsMV-2880F | CCATTGACCTCTTGACAATATTTGT | 1300 |  |  |
| DSMV-4162R | TTTCCTGAACCCACAGCTCCACGTA |  |  |  |
| DSMV-4111F | TACTCCACGAGGATCATGAGTTT | 1000 |  |  |
| DsMV-5100R | ATTTATTTAACAGCATCTCTGACT |  |  |  |
| DsMV-5041F | AGGTTCACAAAATATTAAAGGGAT | 1900 |  |  |
| DSMV-6991R | GCCACCATGGGAATTCCACA |  |  |  |
| DSMV-6936F | TTTTGGAAGCATTGGATATC | 1000 |  |  |
| DsMV-7980R | TTCCGTCTGGGGTCAATATGGGAGT |  |  |  |
| DsMV-7921F | AAATGCTTGATAATTTGTATGCTGA | 2000 |  |  |
| DSMV-9936R | TACCGCGCCAGTGGCGCACT |  |  |  |
| DaMV-CP-F | CGGGATCCGCAGATAATACCGTTGATGC | 900 |  |  |
| DaMV-CP-R | GCGTCGACTTACTGCGGAGATGCTACAC |  |  |  |
| Primers for MVBaV S RNA | | | | |
| S-1-F | AGAGCAATCA GGGTATTAAT TT | 1385 | The 3’- terminal including N gene of MVBaV S RNA | Designed from the contigs |
| S-1-R | TTACTTGAATTCCAAAGTATA |  |  |  |
| S-2-F | ACTCTGATCTATCTGAACAGCA | 825 | IGR |  |
| S-2-R | TCACTCGTTATGGATGATTACG |  |  |  |
| S-3-F | GTAAATCTTA ATGCAAAGAA | 928 | NSs |  |
| S-3-R | TTAGAGCAATCGAGGTAT |  |  |  |
| Primers for MVBaV M RNA | | | | |
| M-1-F | AGAGCAATCG GTGCACGAAT | 1040 | The 3’- terminal including N gene of MVBaV RNA |  |
| M-1-R | CCTTATTTGATTTAAACAAG |  |  |  |
| M-2-F | AGAACGAAGT CGATATTGAT | 730 | 931-1660 | Designed from the contigs |
| M-2-R | GTTTGTTCAGGTTGATCAGGAT |  |  |  |
| M-3-F | TTCCTATGCA CACAGCCAAA | 1210 | 1471-2680 |  |
| M-3-R | ACTGCAAAGATTGATACAAC |  |  |  |
| M-4-F | AGCACATTTG CCTGTACATG | 1260 | 2621-3833 |  |
| M-4-R | TGCGGTGATAAGTCTAGTTT |  |  |  |
| M-5-F | CAGAATACTT GGAGACACAG | 897 | 3811-4707 |  |
| M-5-R | AGAGCAATCAGTGCAACAAT |  |  |  |
| Primers for MVBaV L RNA | | | | |
| 1bp-F | AGAGCAATCGAGCAACAA | 759 | 1-1759 |  |
| 759bp-R | TAATGAAACTATTGAGAAGCC |  |  |  |
| 746bp-F | TCATTGCAAAATGGCTTCTCA | 801 | 746-1526 |  |
| 1526bp-R | CAGAGGAGTTTGAAAACTATC |  |  |  |
| 1388bp-F | GTTGATAAACTATTTGTCC | 966 | 1388-2330 |  |
| 2330bp-R | ATTCAGACCGCAAATGATTGC |  |  |  |
| 2310bp-F | CCATGCTGCATGTTTGCTCAT | 685 | 2310-2970 |  |
| 2972bp-R | GTCAGCTGTTTATGGAATGG |  |  |  |
| L-1-F | ACAGCTGACA TTACAATAAG TC | 1160 | 2961-4210 |  |
| L-1-R | TATACACATCACCATGTTGCTTA |  |  |  |
| L-2-F | ATTGAGTATATACTTAATGC | 1470 | 4011-5480 |  |
| L-2-R | TGATATTAAAGGCTCTGACT |  |  |  |
| L-3-F | ATCCATATC GGACTTTCAT G | 1380 | 5242-6251 |  |
| L-3-R | TCAGGTGTTCCTTATATCAC |  |  |  |
| L-4-F | GAGGTCTCAT GATGTTGATG | 990 | 6391-7380 |  |
| L-4-R | CGAAGAAGCTCATACATCAT |  |  |  |
| L-5-F | ATGCTCCCG TCTTTAATAT C | 1714 | 7212-8926 |  |
| L-5-R | GAAAACGGGGTTGTGGGAGA |  |  |  |
| Primers for TZSV genome | | | | |
| TZSV-NSm-F | ATGTCTCGCATTACTAACGTCCT | 1380 | NSm |  |
| TZSV-NSm-R | GAAATCTAATGTGTTGTCAACATCT |  |  |  |
| TZSV-M-IGRF | GCTAGCATTGGAGTCAGAGATGG | 567 | IGR(903-1469) |  |
| TZSV-M-IGRR | AGGAAACACAAGATGGAAGATTC |  |  |  |
| TZSV-gp-F | ATGAAGAAATACTATCTACCTATCT | 3372 | G protein gene |  |
| TZSV-gp-R | AATATCTAAAGAAAATTGGTAATTC |  |  |  |
| TZSV-NSs-F | ATGTCTACTGCAAAGATGTCTGCTG | 930 | NSs |  |
| TZSV-NSs-R | AGCAGTTTGAACCTTTTCCTCAGAT |  |  |  |
| TZSV-S-IGRF | CTCCGG GGTCAGATCT GGCTGA | 1147 | IGR(1395-2541) |  |
| TZSV-S-IGRR | GAAGCAGATG GAGACTGCAT TC |  |  |  |
| TZSV-N-F | ATGTCTAACGTCCGGAGTTTAACA | 837 | N |  |
| TZSV-N-R | AAAAGACAGATCATTGCTGCTCTT |  |  |  |
| TZSV-L-1F | AGAGCAATCG AGCAACAAAG TA | 1286 | The 5’- terminal of TZSV L RNA |  |
| TZSV-L-1286R | ATTCACTCTCAGCCAGTGAAGT |  |  |  |
| TZSV-L-1192F | GCTTGCATGGCATGCTTCACAAG | 2228 | TZSV L RNA |  |
| TZSV-L-3419R | CATGTCTAACCACCCAGAAGCC |  |  |  |
| TZSV-L-3201F | CATGCATGTTTTCTCTGTATGTC | 2244 |  |  |
| TZSV-L-5444R | GTCACGATGAAGACAATGAGTC |  |  |  |
| TZSV-L-5280F | CTACTGTCAAA GGGTGGCTGT | 1719 |  |  |
| TZSV-L-6998R | GCTCAGACAA CAAGAGTGGT GA |  |  |  |
| TZSV-L-6849F | AC ATGGCCTGTA TCATAGACCT AC | 2069 | The 3’-terminal of TZSV L RNA |  |
| TZSV-L-3end | AGAGCAATCG TGCAACAAAA TAAAG |  |  |  |
|  | | | | |
| INSV-NF | ATGAACAAAGCAAAGATTACCAAG | 789 | N | Hu et al. 2023 |
| INSV-NR | TTAAATAGAGTCATTTTTCCCAA |  |  |  |
